# Blue-Light Hazard of Light-Emitting Diodes Assessed with Gaussian Functions

**DOI:** 10.3390/ijerph18020680

**Published:** 2021-01-14

**Authors:** Stefan Bauer

**Affiliations:** Federal Institute for Occupational Safety and Health (BAuA), 44149 Dortmund, Germany; bauer.stefan@baua.bund.de; Tel.: +49-231-9071-2316

**Keywords:** light-emitting diode (LED), blue-light hazard (BLH), photobiological safety, risk assessment, BLH efficiency, BLH efficacy of luminous radiation, chromaticity coordinates, correlated color temperature (CCT)

## Abstract

The high blue proportion of phosphor-conversion white-light emitting diodes (pc-LEDs), especially of those with higher correlated color temperatures (CCT), raises concern about photochemically induced retinal damages. Although almost all general lighting service LEDs are safe, other applications exist, like spotlights for theatres or at construction sites, that can pose a severe blue-light hazard (BLH) risk, and their photobiological safety must be assessed. Because of required but challenging radiance measurements, a calculative approach can be supportive for risk assessment. It is the aim of this work to exploit Gaussian functions to study LED parameter variations affecting BLH exposure. Gaussian curve approximations for color LEDs, the BLH action spectrum, and the spectral luminous efficiency for photopic vision enabled analytically solving the BLH efficiency, $\eta_{B}$, and the BLH efficacy of luminous radiation, $K_{B,v}$. It was found that sigmoidal functions describe the CCT dependence of $\eta_{B}$ and $K_{B,v}$ for different color LEDs with equal spectral bandwidth. Regarding pc-LEDs, variations of peak wavelengths, intensities, and bandwidths led to linear or parabolic shaped chromaticity coordinate correlations. $\eta_{B}$ and $K_{B,v}$ showed pronounced CCT dependent extrema that might be exploited to reduce BLH. Finally, an experimental test of the presented Gaussian approach yielded its successful applicability for color and pc-LEDs but a minor accuracy for blue and green LEDs.

## 1. Introduction

Semiconductor based light-emitting diodes (LEDs) have an exceptionally successful history, for example, with the blue LED Nobel prize [1], and their importance is still growing not only for general lighting service (GLS) lamps but also for a wide range of modern technologies. Starting with small red or green LEDs for visual signaling, they are now available as huge single LEDs or packaged in large arrays with diverse wavelengths ranging from the ultraviolet and visible to the infrared spectral region. One of the most important milestones, however, was the development of blue-LED chips and thereby white-light emitting diodes.

White light can be generated technically either by superimposing at least two complementary wavelengths with associated power ratios [2] or by an additive mixture (also in multi-channel systems) of the three primary colors red, green, and blue (sometimes also with amber). Today, most common are phosphor-conversion white-light emitting diodes (pc-LEDs) [3] equipped with a blue-LED chip, typically made of indium gallium nitride (InGaN), which excites an yttrium aluminum garnet (YAG) phosphor to yellow fluorescence [4]. However, there are also several other phosphors available commercially that can be applied for pc-LEDs. Combining two (or more) pc-LEDs, i.e. warm and cold white ones, allows an easy manipulation of correlated color temperature (CCT). The high luminous efficiency and the long life up to thousands of hours in conjunction with low costs make white-light emitting diodes the most promising candidate for replacing incandescent or fluorescent lighting; thus, becoming public light source no. 1. Irrespective of all these advantages, there are also concerns about the widespread use of pc-LEDs, especially regarding photochemical injuries caused by the high amount of blue light.

Although the human eye has evolved over millions of years and is well adapted to perceive visible radiation (light, 380–780 nm), exposures to bright light can induce severe damages. Two subtypes of photochemical retinal injuries have been identified, Noell and Ham damage. The latter is usually referred to as blue-light hazard [5], and its relative spectral sensitivity ranges from 300–700 nm with a maximum between 435–440 nm [6]. Light and infrared radiation can also lead to thermal retinal and corneal damages or influence skin health [7]. Other adverse health effects originate from flicker, glare, or circadian rhythm disruption [8]. Up to now, a correlation between chronic light exposure, even below the BLH exposure limit value (ELV), and age-related macular degeneration (AMD) has not been proven convincingly [9,10]. Recently, the absence of red and infrared wavelengths in the electromagnetic spectrum of most pc-LEDs has been discussed with respect to AMD [11].

In general, daily exposures to light from monitors, displays, pc-LEDs, or conventional lighting does not pose any acute BLH risk under “normal” viewing conditions [12,13,14]. However, the BLH ELV may be exceeded, for example, within an eight-hour working day upon viewing sources like stage lights, specialized industrial products for construction sites, or high-power LEDs for optical communication systems [15]. Moreover, the continuing development of semiconductor technology still leads to more and more powerful, energy efficient, and inexpensive light sources. Even though the natural aversion response ensures short exposure durations, t, the BLH ELV is given as effective (BLH weighted) radiance dose, $D_{B}^{\mathrm{EL}}=$ 10^6^ Jm^−2^sr^−1^, for 0.25 s ≤t≤ 10,000 s; thus, it is cumulative. Longer exposure durations, t> 10,000 s, are linked to the effective radiance that is limited to $L_{B}^{\mathrm{EL}}=$ 100 Wm^−2^sr^−1^ [6].

However, radiance measurements can be challenging, especially for non-uniform light sources [16], and the necessary optical instruments are expensive. There exists a standardized approach [17] that regards illuminants, seen by a field-of-view γ< 11 mrad at a 500 lx or a 200 mm distance, as point sources, and therefore allows to measure BLH weighted irradiance considering either $E_{B}\leq$ 100 ${\mathrm{Wm}}^{-2}/t$ (t≤ 100 s) or $E_{B}\leq$ 1 Wm^−2^ (t> 100 s). A calculative method circumventing (ir-) radiance measurements is given by the adaptation of Gaussian functions to the LED signal [18]. Some key optical parameters, for example, noted on the manufacturer’s data sheet, suffice to compute the LED’s BLH weighted radiation emission. It is this article’s aim to simulate color LED and pc-LED emission spectra by means of Gaussian curves in order to analyze effects of different spectral distributions on BLH in detail. In contrast to, for example, descriptions with mathematical series of sine or cosine functions [19,20], the software implementation of Gaussian curves is simple, and well-known antiderivatives exist that can be applied to solve certain convolutions analytically. Aside from these advantages, the Gaussian functions used hereinafter do not account for asymmetric line broadening that can be important, for example, to describe the phosphor’s light emission.

The materials and methods section will present the concept of this Gaussian approach. Although these equations cannot be transferred easily to pc-LEDs, the results section will demonstrate the usefulness of Gaussian functions for a thorough analysis of varying peak wavelengths, bandwidths, and CCTs affecting pc-LEDs’ BLH. An experimental accuracy test later in this work will show that the Gaussian method is sufficiently accurate to describe experimental values for pc-LEDs. Furthermore, having a closer look at the BLH dependence on the above-mentioned variables in conjunction with the CCT can help to develop pc-LEDs with a smaller blue proportion but still preserving the desired light characteristics.

## 2. Materials and Methods

The following sections will explain, in particular, the adaptation of Gaussian functions to LED emission spectra as well as to the BLH weighting function and the spectral luminous efficiency for photopic vision. Their convolutions are described taking biologically effective radiation into account. Two related quantities, the BLH efficiency and the BLH efficacy of luminous radiation, are presented, too. The calculations of chromaticity coordinates and of CCTs, two fundamental colorimetric concepts, are well-established procedures, and their basics are summarized in the Appendix.

### 2.1. LED Emission Spectra

**Figure 1:**
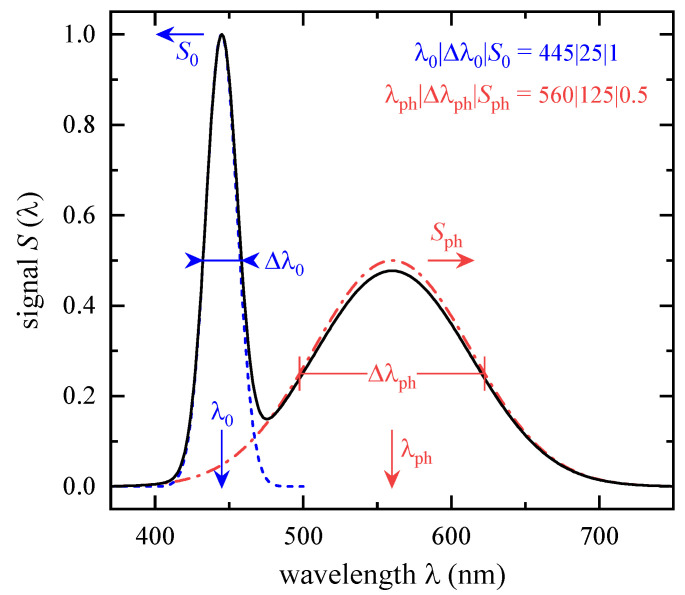
Exemplary signal S(λ) for a color and a phosphor-conversion white-light emitting diode (pc-LED) according to Equation (1). The blue-LED signal (dashed line), centered at the peak wavelength $\lambda_{0}=$ 445 nm with a spectral bandwidth of $\Delta \lambda_{0}=$ 25 nm, is normalized to its maximum, $S_{0}$. The yellowish-green phosphor emission with parameter triple $\lambda_{\mathrm{ph}}\left|\Delta \lambda_{\mathrm{ph}}\right|S_{\mathrm{ph}}=$ 560|125|0.5 is given as dash-dotted line. The addition of both Gaussian curves, subsequently peak normalized, represents a pc-LED (solid line) with x= 0.30, y= 0.31, and $T_{\mathrm{cp}}=$ 7396 K.

A systematic BLH analysis of LED parameter variations can be conducted either by determining experimentally optical measurands like (ir-) radiance or by a calculative approach with the semiconductor’s radiation emission, hereinafter simply referred to as signal S(λ), described mathematically. Based on the fundamental physics of semiconductors, S(λ) can be approximated by a Gaussian function [21],

(1) $$S\left(\lambda \right)=S_{0}\exp\left[\frac{-2{\left(\lambda -\lambda_{0}\right)}^{2}}{{\Delta \lambda }_{0}^{2}}\right]$$

with peak intensity $S_{0}$, wavelength λ, peak wavelength $\lambda_{0}$, and spectral bandwidth $\Delta \lambda_{0}$. For simplicity, S(λ) is not related to a specific physical quantity so that $S_{0}$ is dimensionless. Other mathematical descriptions can also be found in literature, e.g., using a sum of several Gaussian or cosine-power functions [19,20]. Equation (1) is not normalized to an area of 1; thus, the desired spectral bandwidth $\Delta \lambda =1.178\;\Delta \lambda_{0}$ (derived empirically) must be corrected. One example for an LED signal according to Equation (1) is presented in Figure 1. The three Gaussian parameters are written as triple $\lambda_{0}\left|\Delta \lambda_{0}\right|S_{0}$ that is 445|25|1 for the depicted blue-LED light emission. Hereinafter, such single emission line LEDs will be referred to as color LEDs although there is no color perception of the human visual apparatus in the ultraviolet spectral region.

Approximating the YAG phosphor fluorescence by a second Gaussian function according to Equation (1) with parameter triple $\lambda_{\mathrm{ph}}\left|\Delta \lambda_{\mathrm{ph}}\right|S_{\mathrm{ph}}$ allows a mathematical description of some pc-LED emission spectra. Adding both Gaussian functions, $\lambda_{0}\left|\Delta \lambda_{0}\right|S_{0}+\lambda_{\mathrm{ph}}\left|\Delta \lambda_{\mathrm{ph}}\right|S_{\mathrm{ph}}$, and subsequently normalizing the sum results in a characteristic emission spectrum, see Figure 1. It has to be noted that neither a temperature-induced asymmetric line broadening nor a long-wavelength tailoring of the phosphor’s light emission can be considered by Equation (1). The latter is negligible for BLH due to the minor (≤0.001) relative spectral effectiveness of B(λ) for λ≥ 600 nm, but it can have a large effect on the luminous signal, $S_{v}$, weighted by the spectral luminous efficiency for photopic vision, V(λ).

### 2.2. Relative Spectral Effectiveness

Radiometric optical quantities play a minor role in LED safety, but the biologically relevant radiation must be considered. Usually, the emission spectrum of a radiation source is convoluted with a weighting function that describes the relative spectral effectiveness of the biological effect of interest. For this work, the focus is on the BLH action spectrum, B(λ), and the spectral luminous efficiency for photopic vision, V(λ), although there are many other relative spectral effectiveness [22].

#### 2.2.1. Blue-Light Hazard Action Spectrum

**Figure 2:**
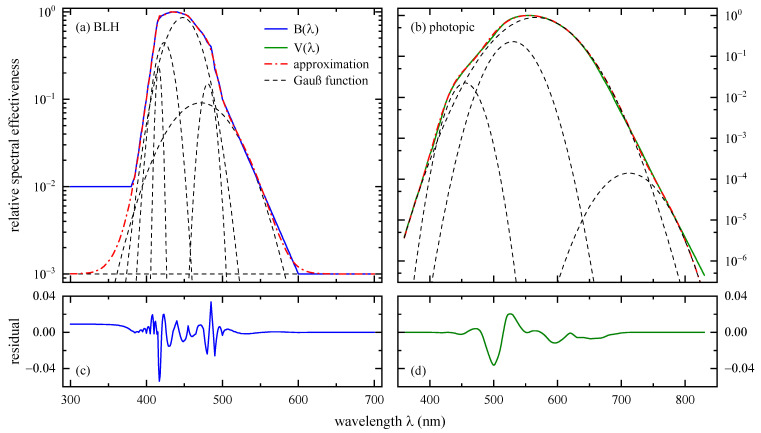
Semi-logarithmic presentation of relative spectral effectiveness for (**a**) the blue-light hazard (BLH) action spectrum, B(λ), and (**b**) the spectral luminous efficiency for photopic vision, V(λ). Gaussian curves according to Equation (2) with fitting parameters given in Table 1 are visualized by dashed lines. Their additive superpositions (dash-dotted lines) approximate B(λ) or V(λ). The wavelength dependent residuals are shown in panels (**c**) and (**d**).

Photochemically induced retinal damage predominantly appears upon exposure to blue light. Therefore, the hazard’s relative spectral effectiveness, the BLH action spectrum [6], has a maximum between 435 nm and 440 nm, see Figure 2. B(λ) also takes wavelengths into account ranging from 300 nm to 700 nm. Derived from a compilation of several experimental studies, no B(λ) equation exists [23]. However, Chaopu et al. [24] successfully approximated the BLH action spectrum by a sum of 5 Gaussian functions parametrized by

(2) $$A\left(\lambda \right)=\underset{k}{\sum }A_{k}\exp\left[\frac{-{\left(\lambda -\lambda_{k}\right)}^{2}}{w_{k}}\right]+C$$

with $A_{k}$ representing a specific maximum relative spectral effectiveness, $\lambda_{k}$ the peak wavelength, $w_{k}$ the spectral bandwidth, and k an index running from 1 to 5. These parameters are reproduced in Table 1. Chaopu et al. [24] found C= 6.737 × 10^−4^ for the constant additive term, but this leads to an underestimation below 380 and above 600 nm. A value of C= 0.001 compensates at least the latter one, see Figure 2a, and thus will be used throughout this work.

#### 2.2.2. Spectral Luminous Efficiency for Photopic Vision

The human eye perceives colors of equal luminous intensity as differently bright with a maximum at λ= 555 nm (green) for daylight (photopic) vision. This sensitivity is reproduced by the spectral luminous efficiency for photopic vision, sometimes briefly referred to as luminosity function, V(λ), see Figure 2b. Hereinafter, the joint version from the International Organization for Standardization and the International Commission on Illumination (ISO/CIE) [25] for a CIE 1931 standard colorimetric 2° observer will be used. However, it must be noted that there is reasonable doubt that this V(λ) does not represent the real human visual perception of brightness [26,27,28].

Chaopu et al. [24] approximated the spectral luminous efficiency for photopic vision, too, but in contrast to B(λ) they applied an asymmetric double sigmoidal function. No successful reproduction of their V(λ) fitting results could be achieved for this work. Instead, in analogy to the approximation of the BLH weighting function, a sum of four Gaussian curves according to Equation (2) was adapted to V(λ), see Figure 2b. The additive constant was set to zero, C= 0. All fitting parameters are listed in Table 1. There are large uncertainties for the fourth fitting curve, in particular for $A_{k}$ and $w_{k}$, indicating that three Gaussian functions might have been sufficiently accurate, too. The coefficient of determination is $R^{2}>$ 0.999. The residual is shown in Figure 2d, and only exhibits two pronounced deviations of −0.04 at 500 nm and of 0.02 at 526 nm.

### 2.3. Signal Weighting and Related Quantities

Both action spectra are necessary for the calculation of biologically effective radiation. The BLH weighted radiation, $S_{B}$, is given by the convolution of the LED’s signal, S(λ), with B(λ), and the luminous signal, $S_{v}$, by convoluting S(λ) with V(λ).

(3) $$S_{B}=\overset{700\;\mathrm{nm}}{\underset{300\;\mathrm{nm}}{\int }}S\left(\lambda \right)\;B\left(\lambda \right)d\lambda$$

(4) $$S_{v}=K_{m}\overset{830\;\mathrm{nm}}{\underset{360\;\mathrm{nm}}{\int }}S\left(\lambda \right)\;V\left(\lambda \right)d\lambda$$

The constant factor $K_{m}=$ 683 lmW^−1^ represents the maximum spectral luminous efficacy. The integration limits 360 to 830 nm have been chosen according to the recommendation of ISO/CIE 11664-1 to obtain tristimulus values [25], but other wavelength ranges like 380 to 780 nm are also quite common. Because of the low relative spectral sensitivity (<5 × 10^−5^) of V(λ) below 380 nm and above 780 nm, a change between both integration limits will have a minor or even negligible effect on $S_{v}$.

Based on $S_{B}$ and $S_{v}$, two additional quantities can be applied for the risk assessment of LEDs. The dimensionless BLH efficiency, $\eta_{B}$, standardized by the International Electrotechnical Commission (IEC) [29], reflects the ratio of $S_{B}$ to the LED’s radiometric (unweighted) signal within the wavelength range from 300 to 700 nm.

(5) $$\eta_{B}=S_{B}{\left[\overset{700\;\mathrm{nm}}{\underset{300\;\mathrm{nm}}{\int }}S\left(\lambda \right)d\lambda \right]}^{-1}$$

Theoretically assuming very large bandwidths $\Delta \lambda_{0}$, S(λ) can be approximated by 1, and the numerator in Equation (5) is the integral of the BLH weighting function, only, yielding a constant value of 68.97 nm. With the denominator’s value being equal to 700 nm − 300 nm = 400 nm, a convergence point exists, $\underset{\Delta \lambda_{0}⟶\infty }{\lim}\eta_{B}=$ 0.17.

The second risk assessment quantity is the BLH efficacy of luminous radiation, also standardized by IEC [29], that is the ratio of the BLH weighted to the luminous signal.

(6) $$K_{B,v}=\frac{S_{B}}{S_{v}}$$

$K_{B,v}$ is often presented as a function of correlated color temperature, $T_{\mathrm{cp}}$, because many white-light sources have comparable $K_{B,v}$ values for equal $T_{\mathrm{cp}}$ [29]. Due to $K_{m}$ the unit of $K_{B,v}$ is watt per lumen, Wlm^−1^. Similar to the considerations made for $\eta_{B}$ very large bandwidths in Equation (6) lead to a convergence point located at $K_{B,v}=$ 9.5 × 10^−3^ Wlm^−1^.

## 3. Results and Discussion

Combining the adaptations of Gaussian functions to LED emission spectra as well as to the BLH weighting function and the spectral luminous efficiency for photopic vision allows the determination of analytical solution for $S_{B}$ and $S_{v}$; thus, of $\eta_{B}$ and $K_{B,v}$, at least for color LEDs. Regarding pc-LEDs, the effect of several parameter variations on the BLH can be studied by Gaussian curve fitting. In the following, these results will be presented and discussed.

### 3.1. Color LEDs

#### 3.1.1. Analytical Solutions

The BLH weighted and the luminous signal, $S_{B}$ and $S_{v}$, Equations (3) and (4), respectively, can be solved analytically for color LEDs because S(λ) as well as both weighting functions being parametrized by exponential functions with a quadratic term as exponent. Multiplying the LED signal with B(λ) or V(λ) yields another Gaussian function, and writing the quadratic exponent in its long form results in the antiderivative

(7) $$P_{i}\int^{\;}\exp\left(-p_{1}\lambda^{2}+p_{2}\lambda +p_{3}\right)d\lambda =\frac{\sqrt{\pi }}{2\sqrt{p_{1}}}\;\exp\left(\frac{p_{2}^{2}}{4p_{1}}+p_{3}\right)\mathrm{erf}\left(\sqrt{p_{1}}\lambda -\frac{p_{2}}{2\sqrt{p_{1}}}\right)$$

with parameters $p_{1}=\frac{2}{\Delta \lambda_{0}^{2}}+\frac{1}{w_{k}}$, $p_{2}=\frac{4\lambda_{0}}{\Delta \lambda_{0}^{2}}+\frac{2\lambda_{k}}{w_{k}}$, and $p_{3}=-\frac{2\lambda_{0}^{2}}{\Delta \lambda_{0}^{2}}-\frac{\lambda_{k}^{2}}{w_{k}}$. The uppercase $P_{i}$ stands either for $S_{0}A_{k}$ (k= 1 to 5, BLH) or $K_{m}S_{0}A_{k}$ (k= 6 to 9, luminous signal). The error function can be approximated by erf≈±1, yielding a factor of 2 in Equation (7), for the given integration limits (300 to 700 nm and 360 to 830 nm) in conjunction with bandwidths $\Delta \lambda_{0}$ smaller than ca. 500 nm covering all relevant color LEDs. Later in this work, even bigger $\Delta \lambda_{0}$ will be regarded for theoretical purposes, and the exact values of the error function must be taken into account. The integral that originates from the constant term C in Equation (2) yields $\sqrt{\pi /2}CS_{0}\Delta \lambda_{0}$ when setting the action spectra parameter $\lambda_{k}$ and $w_{k}$ in the antiderivative expression in Equation (7) to zero, $A_{k}=$ 1, and applying the error function approximation. Overall, the analytical solution for the blue-light hazard weighted color-LED signal given in Equation (3) can be written as

(8) $$S_{B}\left(\lambda_{0},\Delta \lambda_{0}\right)=S_{0}\underset{k}{\sum }A_{k}\sqrt{\frac{\pi \;w_{k}\;\Delta \lambda_{0}^{2}}{2w_{k}+\Delta \lambda_{0}^{2}}}\;\exp\left[\frac{-2{\left(\lambda_{0}-\lambda_{k}\right)}^{2}}{2w_{k}+\Delta \lambda_{0}^{2}}\right]+\sqrt{\frac{\pi }{2}}CS_{0}\Delta \lambda_{0}$$

The result for the convolution of S(λ) with V(λ) in Equation (4), $S_{v}\left(\lambda_{0},\Delta \lambda_{0}\right)$, looks similar to Equation (8) but with k= 6 to 9, see Table 1, C= 0, and the additional factor $K_{m}$.

At first glance, it seems to be straightforward to solve $S_{B}$ or $S_{v}$ for pc-LEDs: instead of being the product of two Gaussian functions the integrand is given by the addition of the blue-LED signal and the phosphor fluorescence both multiplied by the weighting function. Splitting up this integral into two summands allows an application of Equation (7). However, this is wrong. The pc-LED signal must be peak normalized in order to ensure comparability of the results; thus, the two Gaussian functions must stay together. This normalization is bisected, depends on the later signal maximum located at $\lambda_{0}$ or $\lambda_{\mathrm{ph}}$, and is a function of all pc-LED parameters. Consequently, the integrands in Equations (3) and (4) become much more complex, and the antiderivative in Equation (7) can no longer be applied. One approach to solve $S_{B}$ or $S_{v}$ analytically for pc-LEDs could be the use of Gaussian functions that are normalized to their area [30,31]; however, with a much more complex antiderivative.

Concerning the BLH efficiency of color LEDs, the denominator in Equation (5) can be solved by means of Equation (7) resulting in $\sqrt{\pi /2}S_{0}\Delta \lambda_{0}$. In conjunction with Equation (8), the BLH efficiency can be written as

(9) $$\eta_{B}=\underset{k}{\sum }A_{k}\sqrt{\frac{2w_{k}}{2w_{k}+\Delta \lambda_{0}^{2}}}\;\exp\left[\frac{-2{\left(\lambda_{0}-\lambda_{k}\right)}^{2}}{2w_{k}+\Delta \lambda_{0}^{2}}\right]+C$$

Inserting very large bandwidths, $\eta_{B}$ approaches C showing the restricted applicability of the error function approximation. It is worth mentioning, that some criticism on luminous efficacy, a similar quantity to $\eta_{B}$, was published by Houser [32]. Note that the term efficiency refers to a dimensionless quantity whereas efficacies have units like watt per lumen or vice versa in the field of optics. It is possible to solve the equation for the BLH efficacy of luminous radiation analytically as given by Equation (6); however, dividing a sum of five by a sum of four Gaussian functions does not result in a simple mathematical expression. Hence, all $K_{B,v}$ values for color LEDs were calculated applying Equation (8), subsequently dividing $S_{B}$ by $S_{v}$.

#### 3.1.2. Weighted LED Signals

**Figure 3:**
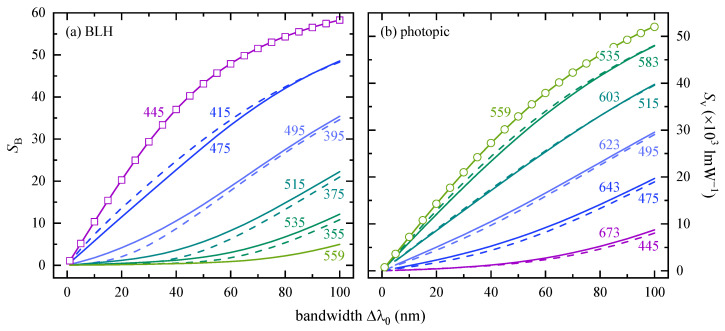
Radiation emission from color LEDs weighted with (**a**) the blue-light hazard action spectrum and (**b**) the spectral luminous efficiency for photopic vision, $S_{B}\left(\Delta \lambda_{0}\right)$ and $S_{v}\left(\Delta \lambda_{0}\right)$ according to Equation (8), respectively, depending on spectral bandwidth, $\Delta \lambda_{0}$. The peak wavelengths, $\lambda_{0}$, are given as numbers in nm, and vary in (**a**) with regard to 445 nm in steps of −30 nm or −20 nm (dashed lines) and 20 nm or 30 nm (solid lines). Open symbols represent discrete calculations based on Equations (3) or (4). Whenever possible, the line colors from (**a**) were also used in (**b**) with an equal ±20 nm or ±30 nm peak wavelength difference except for $\lambda_{0}=$ 559 nm for that the maximum $S_{v}\left(\Delta \lambda_{0}\right)$ values were found. Due to $K_{m}$, the $S_{v}$ ordinate in panel (**b**) has the unit lmW^−1^ and is divided by a factor of 1000.

Results of BLH weighted radiation emissions, $S_{B}\left(\lambda_{0},\Delta \lambda_{0}\right)$, and luminous signals, $S_{v}\left(\lambda_{0},\Delta \lambda_{0}\right)$, both derived analytically by means of Equation (8), are presented in Figure 3 as a function of spectral bandwidth ranging from 1 to 100 nm and for several peak wavelengths, 355 nm $\leq \lambda_{0}\leq$ 673 nm. The highest potential for photochemically induced retinal damage is found for color LEDs with $\lambda_{0}=$ 444.8 nm. This wavelength is close to the maximum BLH relative spectral effectiveness between 435 to 440 nm, but it does not coincide due to the asymmetric line shape of B(λ). Having the error function approximation in mind, an exemplary comparison of the analytical (solid line) with the discrete calculation (open squares) according to Equations (3) and (8) is depicted for $\lambda_{0}=$ 444.8 nm, and all percentage deviations are below 0.35%. Shifting the peak wavelengths in steps of ±30 nm leads to an overall $S_{B}\left(\lambda_{0},\Delta \lambda_{0}\right)$ decrease. While both BLH weighted signals are close to each other for $\Delta \lambda_{0}\geq$ 80 nm, $S_{B}\left(\lambda_{0}=415\;\mathrm{nm}\right)$ > $S_{B}\left(\lambda_{0}=475\;\mathrm{nm}\right)$ for smaller bandwidths. However, this trend reverses upon further ±20 nm peak wavelength shifts. Note that UV LEDs, i.e. those with $\lambda_{0}=$ 355, 375, 395 and maybe even 415 nm, can pose a non-negligible erythemal hazard or can harm the exterior eye media, depending on their spectral bandwidth, $\Delta \lambda_{0}$.

The human visual apparatus recognizes a green LED with $\lambda_{0}=$ 559.1 nm as the brightest one with the maximum $S_{v}\left(\Delta \lambda_{0}\right)$, see Figure 3b, but with $S_{B}$ < 5 a.u. it can hardly be associated with a BLH risk disregarding exposure distance and duration. Again, the accuracy of Equation (8) (solid line) compared to 4 (open circles) was evaluated, and the percentage deviations are below ±0.24%. Similar to the findings for the BLH weighted signal, the shift to the smaller peak wavelength $\lambda_{0}=$ 535 nm is accompanied by a slightly higher luminous signal in terms of $S_{v}\left(\lambda_{0}=583\;\mathrm{nm}\right)$, the curves for $\lambda_{0}=$ 515 nm and $\lambda_{0}=$ 603 nm nearly match each other, and a further increase of $\lambda_{0}$ is associated with higher luminous signals (solid lines) than compared to their short wavelength analogues (dashed lines).

#### 3.1.3. Processed BLH Quantities

**Figure 4:**
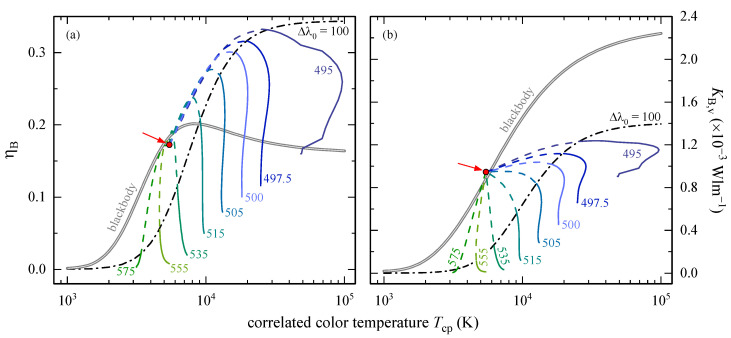
(**a**) BLH efficiency, $\eta_{B}$, and (**b**) BLH efficacy of luminous radiation, $K_{B,v}$, of color LEDs depending on bandwidth, $\Delta \lambda_{0}$, and plotted versus correlated color temperature (CCT), $T_{\mathrm{cp}}$, on a logarithmic abscissa. The peak wavelengths, $\lambda_{0}$, are given as numbers in nanometer. Solid lines represent a 1 nm $\leq \Delta \lambda_{0}\leq$ 100 nm bandwidth variation. Further increasing $\Delta \lambda_{0}$ up to 1000 nm (dashed lines) leads to (**a**) $\eta_{B}=$ 0.17 and (**b**) $K_{B,v}=$ 9.5 × 10^−4^ Wlm^−1^ convergence points (full circles highlighted by arrows) at $T_{\mathrm{cp}}=$ 5463 K with x=y= 0.33. The dash-dotted lines parametrize $\eta_{B}$ and $K_{B,v}$ for $\Delta \lambda_{0}=$ 100 nm according to the five-parameter logistic function in Equation (10). The blackbody radiator’s $\eta_{B}\left(T_{\mathrm{cp}}\right)$ and $K_{B,v}\left(T_{\mathrm{cp}}\right)$ are included as double solid lines. Note the steps in both $\lambda_{0}=$ 495 nm curves at 50,000 K that appear because of the parameter change in Equation (A6) (see Appendix A).

The CCT dependence of an LED’s BLH can be analyzed by means of the dimensionless BLH efficiency, $\eta_{B}$, and the BLH efficacy of luminous radiation, $K_{B,v}$, see Equations (6) and (9), respectively. Figure 4a shows $\eta_{B}\left(\lambda_{0},\Delta \lambda_{0},T_{\mathrm{cp}}\right)$ for several color LEDs. The CCT was calculated either with Equations (15) or (17) (see Appendix). In accordance with the findings for $S_{B}\left(\lambda_{0},\Delta \lambda_{0}\right)$, see Figure 3a, shorter peak wavelengths are associated with a higher BLH potential, now expressed in terms of $\eta_{B}$, as the denominator of Equation (5), approximated by $\sqrt{\pi /2}S_{0}\Delta \lambda_{0}$, does not depend on $\lambda_{0}$; thus, having the same value for all peak wavelengths of interest. However, this approximation is restricted to those $\lambda_{0}$ that are not in the direct vicinity of the integration limits, 300 and 700 nm. For example, the percentage deviation of the analytical to the discrete $\eta_{B}$ result, Equation (9) with regard to Equation (5), respectively, for $\lambda_{0}=$ 395 nm is still better than 4.5% but becomes worse for shorter wavelengths.

A $\Delta \lambda_{0}$ increase from 1 to 100 nm (solid lines) only has a minor effect on the CCT for greenish-light LEDs like $\lambda_{0}=$ 515 nm for that $T_{\mathrm{cp}}$ is within 9100 to 9700 K. This behavior changes for shorter peak wavelengths and is most prominent for $\lambda_{0}=$ 495 nm. Note that the step in $\eta_{B}\left(\lambda_{0}=495\;\mathrm{nm}\right)$ at 50,000 K originates from the switch between two different CCT regimes in Equation (A6) (see Appendix A). For theoretical bandwidths up to 1000 nm (dashed lines), all BLH efficiencies converge towards $\eta_{B}=$ 0.17 at $T_{\mathrm{cp}}=$ 5463 K (full circle), see section Signal Weighting and Related Quantities, that is close to the blackbody radiator’s BLH efficiency (double line). The signal of such an LED is equally distributed, S(λ)≈ 1, resembling CIE’s standard illuminant E with x=y= 0.33.

For any constant bandwidth, the peak wavelength dependent BLH efficiency can be described empirically by an s-shaped five-parameter logistic function according to

(10) $$\eta_{B}=\eta_{\infty }{\left[1+{\left(\frac{T_{0}}{T_{\mathrm{cp}}}\right)}^{h}\right]}^{-s}$$

The parameter $\eta_{\infty }$ describes a saturation $\eta_{B}$ value for infinitely high $T_{\mathrm{cp}}$, $T_{0}$ is the CCT at which the curvature changes (inflection point), h reflects the steepness of the curve (hill slope), and s is the asymmetry parameter regarding $T_{0}$ (s= 1 is equal to no asymmetry). The fifth parameter that is the minimum asymptotic value is zero; hence, it is not present in Equation (10). Choosing $\Delta \lambda_{0}=$ 100 nm as an example, the dash-dotted line in Figure 4a is a fit to $\eta_{B}\left(\lambda_{0},\;T_{\mathrm{cp}}\right)$ with $\eta_{\infty }=$ 0.344 ± 0.004, $T_{0}=$ (5990 ± 612) K, h= 2.5 ± 0.1, and s= 1.7 ± 0.3. These parameters vary with $\Delta \lambda_{0}$ (not shown), but a thorough analysis is beyond the scope of the present article.

In general, similar trends can be observed for the BLH efficacy of luminous radiation, $K_{B,v}$, calculated according to Equation (6), see Figure 4b: higher $K_{B,v}$ values for smaller peak wavelengths (with $\Delta \lambda_{0}=$ const.), a weak CCT dependence on spectral bandwidth of greenish-light LEDs for $\Delta \lambda_{0}\leq$ 100 nm, and a convergence point $K_{B,v}=$ 9.5 × 10^−4^ Wlm^−1^ at the same CCT, $T_{\mathrm{cp}}=$ 5463 K; however, this time located slightly above $K_{B,v}\left(T_{\mathrm{cp}}\right)$ of the Planckian radiator. Again, the peak wavelength dependence of $K_{B,v}$ can be parametrized by a sigmoidal function according to Equation (10) with $K_{\infty }=$ (1.41 ± 0.03) × 10^−3^ Wlm^−1^, $T_{0}=$ (6300 ± 1171) K, h= 2.0 ± 0.2, and s= 2.4 ± 0.7 for constant $\Delta \lambda_{0}=$ 100 nm. The inflection point and the asymmetry parameter coincide within their uncertainty ranges with those for $\eta_{B}\left(\lambda_{0},\;T_{\mathrm{cp}}\right)$, but no conclusions can be drawn without further analysis. Note that the blackbody radiator’s BLH efficacy of luminous radiation can be described itself by Equation (10) with $K_{\infty }=$ (2.290 ± 0.001) × 10^−3^ Wlm^−1^, $T_{0}=$ (1543 ± 41) K, h= 1.303 ± 0.004, and s= 5.4 ± 0.1.

### 3.2. Phosphor Conversion White LEDs

#### 3.2.1. Parameter Variation Limits

A calculative BLH analysis of pc-LEDs requires a closer look at the meaning of the term “white” itself in order to get limits for the chromaticity coordinates; thus, for the parameter triples. However, defining a light source’s whiteness is a complex subject that can depend on, for example, the perception of brightness and tint, or viewing mode and viewing medium [33]. There exist two international standards [34,35] that can be applied to determine the so-called CIE whiteness under illumination with D65 (outdoor daylight) and C (indoor illumination conditions), but they are only intended for reflecting surfaces like paper. Based on the chromaticity coordinate differences between emitter and reflector, this concept cannot be transferred to illuminants. Wyszecki and Stiles [2] reported about two complementary wavelengths with associated power ratios whose additive superposition is perceived as white light, but their work refers to rather monochromatic or at least narrow band emission lines; thus, it is not applicable for pc-LEDs.

As a consequence, a rather pragmatic approach is used hereinafter to get variation limits for the Gaussian parameter triples $\lambda_{0}\left|\Delta \lambda_{0}\right|S_{0}$ and $\lambda_{\mathrm{ph}}\left|\Delta \lambda_{\mathrm{ph}}\right|S_{\mathrm{ph}}$. The starting point is a typical pc-LED spectrum with its blue InGaN emission between 440 to 470 nm ($\Delta \lambda_{0}\sim$25 nm) and the broad excitation ($\Delta \lambda_{\mathrm{ph}}\sim$150 nm) of a YAG phosphor at wavelength maxima 550 nm $\leq \lambda_{\mathrm{ph}}\leq$ 600 nm [3,4,15,21]. In view of the worst-case peak wavelengths for $S_{B}$ and $S_{v}$ of color LEDs, 444.8 and 559.1 nm, respectively, see Figure 3, $\lambda_{0}=$ 445 nm and $\lambda_{\mathrm{ph}}=$ 560 nm are selected as initial parameters. Spectral bandwidths are chosen to be $\Delta \lambda_{0}=$ 25 nm and $\Delta \lambda_{\mathrm{ph}}=$ 125 nm, and the peak ratio is set to $S_{0}:S_{\mathrm{ph}}=$ 1:0.5. Overall, white light is emitted with x= 0.30, y= 0.31, and $T_{\mathrm{cp}}=$ 7396 K.

Additionally, it is demanded that the chromaticity coordinates of all calculated pc-LED emission spectra are in vicinity to (x,y) of the Planckian locus ranging from 3200 to 40,000 K and to the white points of several CIE standard illuminants. Therefore, the chromaticity diagram is limited to 0.23 ≤(x,y)≤ 0.43 with equal distances to the white point x=y= ⅓. Yamada et al. [36] found comparable values that are (0.23, 0.22) for bluish-white and (0.38, 0.43) for yellowish-white YAG phosphors. It is important to note that the present article focuses on theoretical considerations, and that the following parameter variations are intended to demonstrate general trends, not all of them being practically relevant.

#### 3.2.2. Chromaticity Coordinates

**Figure 5:**
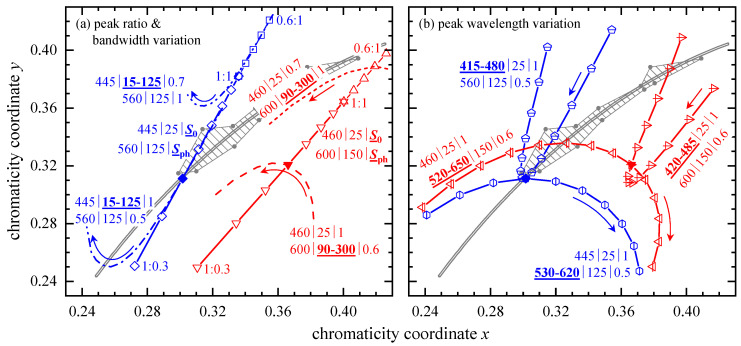
Chromaticity diagrams for calculated pc-LED emission spectra. Bold and underlined characters visualize the varied parameters. (**a**) peak ratio variation, $S_{0}:S_{\mathrm{ph}}$, for (i) 445|25 with 560|125 and either 0.6 $\leq S_{0}\leq$ 1, $S_{\mathrm{ph}}=$ 1 (open squares) or 0.3 $\leq S_{\mathrm{ph}}\leq$ 1, $S_{0}=$ 1 (open diamonds) as well as for (ii) 460|25 with 600|150 and equal $S_{0}:S_{\mathrm{ph}}$ changes (triangles up and down). In the same panel, bandwidth variations are depicted regarding (i) with 15 nm $\leq \Delta \lambda_{0}\leq$ 125 nm and (ii) with 90 nm $\leq \Delta \lambda_{\mathrm{ph}}\leq$ 300 nm. (**b**) Peak wavelength shifts were investigated on the basis of two fixed parameter triple combinations, 445|25|1 with 560|125|0.5 (full diamond) and 460|25|1 with 600|150|0.6 (full triangle down), highlighted in both panels. Overall, solid lines are linear interpolations drawn to guide the eye. Arrows indicate increasing bandwidth or peak wavelength. The hatched area is bordered by the white points (full circles) of the CIE 1931 2° standard illuminants A, B, C, D55, D75, E, F2 to F6, and F9 to F12. Additionally, the Planckian locus, ranging from 3200 K to 40,000 K, is depicted as double solid line.

In a first step, the peak intensities, $S_{0}$ and $S_{\mathrm{ph}}$, were varied for 445|25 and 560|125. The resulting (x,y), calculated according to Equation (A2) in the Appendix, are presented in Figure 5a as open squares and diamonds and show a perfect linear x-y-correlation ($R^{2}=$ 1). The same is true for a second example with parameters 460|25 and 600|150 (open triangles). Note that these peak ratio variations, $S_{0}:S_{\mathrm{ph}}$, do not produce “ideal” pc-LEDs but have red, green, or blue tints with different whiteness [37].

A change of spectral bandwidths leads to parabolic shaped chromaticity coordinates. These parabolas are opened upwards for 15 nm $\leq {\Delta \lambda }_{0}\leq$ 125 nm (dash-dotted and dash-double-dotted line) and downwards for 90 nm $\leq {\Delta \lambda }_{\mathrm{ph}}\leq$ 300 nm (dashed and dotted lines). Higher bandwidths (visualized by arrows) are accompanied by smaller x values. The chromaticity coordinates that originate from a ${\Delta \lambda }_{0}$ decrease converge to those of the peak ratio variation, in contrast to (x,y) upon varying ${\Delta \lambda }_{\mathrm{ph}}$.

Two combinations of parameter triples, 445|25|1 with 560|125|0.5 (full diamond) and 460|25|1 with 600|150|0.6 (full upside-down triangle), see Figure 5b, represent the initial LEDs to study the effect of peak wavelength variations on chromaticity coordinates. Changing $\lambda_{0}$ in both sets results in narrow parabolas that are opened upwards (horizontally dashed pentagons and triangles) whereas variations of the phosphor emission peak wavelengths, $\lambda_{\mathrm{ph}}$, yield broad curved x-y-correlations being opened downwards (vertically dashed hexagons and triangles).

#### 3.2.3. CCT Dependent BLH

**Figure 6:**
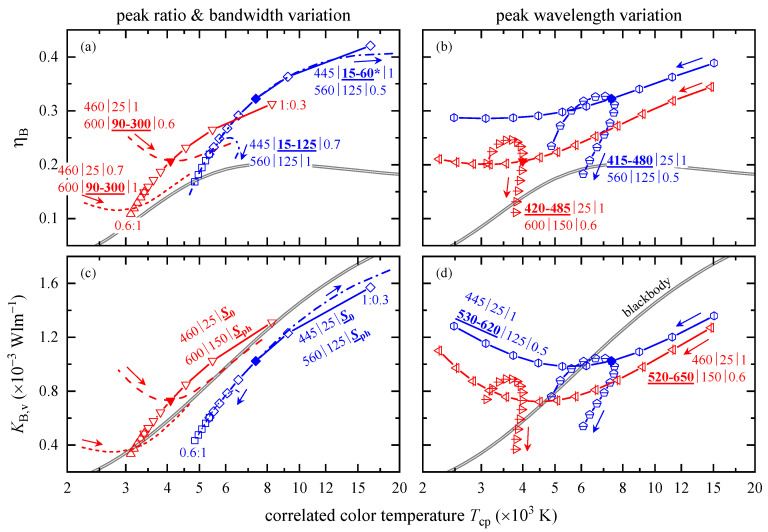
(**a**,**b**) BLH efficiency, $\eta_{B}$, and (**c**,**d**) BLH efficacy of luminous radiation, $K_{B,v}$, as a function of $T_{\mathrm{cp}}$ (logarithmic abscissa), for pc-LED. Panels (**a**,**c**) visualize effects of peak ratio and bandwidth variations, whereas panels (**b**,**d**) focus on peak wavelength variations. The varied parameters are indicated by bold, underlined characters. The same lines and symbols were used as for the chromaticity diagrams, see Figure 5. The asterisk at $\Delta \lambda_{0}$ in panel (**a**) marks the bandwidth reduction from 125 to 60 nm. Solid lines between the data points (symbols) are linear interpolations drawn to guide the eye. Arrows indicate increasing bandwidth or peak wavelength. The double solid lines visualize the blackbody radiator’s $\eta_{B}\left(T_{\mathrm{cp}}\right)$ and $K_{B,v}\left(T_{\mathrm{cp}}\right)$.

Knowledge about chromaticity coordinates and therefore of parameter variation limits is essential in order to study efficiently the BLH potential of pc-LEDs. Based on $S_{B}$ and $S_{v}$ results (not shown), according to Equations (3) and (4), with parameters equal to those given Figure 5, BLH efficiencies and BLH efficacies of luminous radiation were calculated and are depicted in Figure 6. As expected, varying $S_{0}$ from 0.6 to 1 (triangles, squares) and $S_{\mathrm{ph}}$ from 1 to 0.3 (upside-down triangles, diamonds), see panels (a) and (c), increases $\eta_{B}$ and $K_{B,v}$ for both parameter triples accompanied by a shift to higher CCT, because of the growing relative strength of the blue signal. This peak intensity variation demonstrates that the same CCT can be achieved for pc-LEDs but with a different BLH risk, for example, at $T_{\mathrm{cp}}\approx$ 5500 K with a $K_{B,v}$ ratio of ~1.6.

Changes in ${\Delta \lambda }_{0}$, dash-dotted and dash-double-dotted lines in panels (a) and (c), lead to strong overlaps with the $\eta_{B}$ and $K_{B,v}$ curves of the peak ratio variations; thus, having a similar effect on the BLH risk. Note that ${\Delta \lambda }_{0}$ is restricted to 60 nm (instead of 125 nm, highlighted by an asterisk) for the peak ratio $S_{0}:S_{\mathrm{ph}}=$ 1:0.5 in order to reduce the maximum $T_{\mathrm{cp}}$ and to guarantee visual clarity. In contrast, changing the bandwidth of the phosphors excitation leads to parabola-like shaped curves (dashed and short-dashed lines) that are opened upwards. It is not intuitive that smaller $\Delta \lambda_{\mathrm{ph}}$ values are associated with higher BLH risks located at warmer CCTs. The minima occurring could be exploited, in combination with other parameter variations and if technically realizable, to reduce the BLH risk from pc-LEDs while keeping the desired light characteristic, i.e. CCT.

Figure 6b,d depict both peak wavelength variations and their induced changes in $\eta_{B}$ and $K_{B,v}$. Increasing $\lambda_{\mathrm{ph}}$ (vertically dashed symbols) reduces $\eta_{B}$ over a wide range of decreasing $T_{\mathrm{cp}}$, whereas the related BLH efficacies of luminous radiation show pronounced minima. Regarding these $\lambda_{\mathrm{ph}}$ effects, variations of $\lambda_{0}$ take place on a comparably small $T_{\mathrm{cp}}$ range, and the CCT first increases followed by a decrease for higher peak wavelengths. Again, the curved nature of $\eta_{B}$ and $K_{B,v}$, depending either on peak wavelength $\lambda_{0}$ or $\lambda_{\mathrm{ph}}$, might be used to minimize the LED’s BLH risk.

### 3.3. Experimental Accuracy Test

The analytical solutions for $\eta_{B}$ and $K_{B,v}$ of color LEDs, given by Equations (8) and (9), as well as the related formulae for pc-LEDs, Equations (5) and (6), are tested experimentally regarding their accuracies. Therefore, the spectral irradiances, E(λ), of a non-representative selection of color and pc-LEDs, listed in Table 2, were measured according to the alternative method described in IEC 62471 [17] by a thermoelectrically cooled BTS2048-VL-TEC spectroradiometer (Gigahertz-Optik, Türkenfeld, Germany) within 350 to 1050 nm at a distance of 20 cm. Note that there is an inherent systematic error due to the lower wavelength limit, but the BLH action spectrum is only 0.01 below 350 nm, see Figure 2a. Most of the examined LEDs are smaller than or close to 2 mm diameter or edge length; thus, they are seen by a field-of-view γ≈ 11 mrad and can be regarded as small sources with respect to IEC 62471. The LED reflector from Paulmann, j= 37, is by far the largest LED with a diameter of ~15 mm and does not fulfill the small source criterion. The described experimental setup allows the calculation of spectral radiances from the measured irradiances, $L\left(\lambda \right)=E\left(\lambda \right)\Omega^{-1}$, via the solid angle; however, Ω is irrelevant for the discussion of $\eta_{B}$ and $K_{B,v}$ values as it reduces itself from these quotients, see Equations (5) and (6).

As already stated above, the use of Gaussian functions does not take asymmetrically shaped LED emissions into account. However, the center wavelength

(11) $$\lambda_{c}=\frac{\int^{\;}\lambda \;E\left(\lambda \right)\;d\lambda }{\int^{\;}E\left(\lambda \right)\;d\lambda }$$

does consider asymmetry, at least to some extent, and therefore will replace $\lambda_{0}$ of color LEDs in Equations (8) and (9). $\lambda_{c}=\lambda_{0}$ for symmetrically shaped Gaussian curves, and the difference $\lambda_{c}-\lambda_{0}$ can be used to visualize the degree of asymmetry.

**Figure 7:**
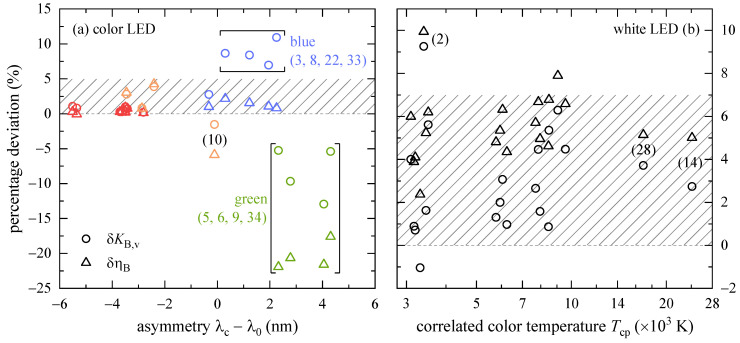
Percentage deviations, $\delta \eta_{B}$ and $\delta K_{B,v}$, for BLH efficiencies and BLH efficacies of luminous radiation, respectively, that were calculated according to Equations (8) and (9) (color LEDs) as well as Equations (5) and (6) (pc-LEDs). The percentage deviations were determined with regard to the measured values of those LEDs listed in Table 2. (**a**) $\delta \eta_{B}$ and $\delta K_{B,v}$ as a function of asymmetry. The symbols are colored with respect to the LED’s chromaticity. (**b**) Semi-logarithmic presentation of $\delta \eta_{B}$ and $\delta K_{B,v}$ for pc-LEDs as a function of $T_{\mathrm{cp}}$. Numbers in brackets refer to the running index j. In both panels, horizontally dashed lines represent zero percentage deviation, and the hatched areas visualize (**a**) 0–5% and (**b**) 0–7% deviation.

The percentage deviations of the analytical expressions for the BLH efficiencies and BLH efficacies of luminous radiation, $\delta \eta_{B}$ and $\delta K_{B,v}$, with respect to their corresponding experimental values are presented in Figure 7a for color LEDs. Those with red, orange, and yellow center wavelengths (except for j= 10), see Table 2, have negative asymmetries, and $\delta \eta_{B}$ and $\delta K_{B,v}$ are within 0 to 5%. For most tested blue LEDs (j= 3, 8, 22, and 33), however, $\delta K_{B,v}>$ 5% with positive asymmetry. A closer look at the relevant data reveals that the analytical luminous intensity, $S_{v}$ according to Equation (8), that is close to zero, is more than 10% smaller than the measured non-zero illuminance (due to stray light, dark current, etc.) resulting in higher $\delta K_{B,v}$ values. $\delta \eta_{B}$ remains unaffected and only has a minor deviation (<2.2%). Similarly, for all green LEDs (j= 5, 6, 9, and 34) as well as for the amber colored one (j= 10), the analytical BLH weighted signals, $S_{B}$ according to Equation (8), are about 15 to 25% smaller than compared to the experimental values yielding $\delta K_{B,v}<$ −5% and $\delta \eta_{B}<$ −17%.

Analyzing the percentage deviations for pc-LEDs revealed no dependencies neither on peak wavelengths, $\lambda_{0}$ and $\lambda_{\mathrm{ph}}$ (not shown), bandwidths, $\Delta \lambda_{0}$ and $\Delta \lambda_{\mathrm{ph}}$ (not shown), phosphor concentrations, $S_{\mathrm{ph}}$ (not shown), nor on correlated color temperature, $T_{\mathrm{cp}}$, see Figure 7b. However, $\delta \eta_{B}$ and $\delta K_{B,v}$ are positive and within 0 to 7%. LEDs j= 14 and 28 have small $S_{\mathrm{ph}}$ values, see Table 2, associated with high CCT values.

## 4. Conclusions

The adaptation of Gaussian functions to color LED emission spectra, to the BLH action spectrum, and to the spectral luminous efficiency for photopic vision allowed to derive analytical solutions for both weighted LED signals, $S_{B}$ and $S_{v}$, for the BLH efficiency, $\eta_{B}$, and the BLH efficacy of luminous radiation, $K_{B,v}$; however, the latter having no simple mathematical expression. Analyzing the peak wavelength dependence showed that photochemically induced retinal damage is highest for a blue LED with $\lambda_{0}=$ 444.8 nm, and that a green LED with $\lambda_{0}=$ 559.1 nm is perceived as the brightest one. With increasing bandwidths, ${\Delta \lambda }_{0}$, all color LEDs converge towards $\eta_{B}=$ 0.17 and $K_{B,v}=$ 9.5 × 10^−4^ Wlm^−1^ at a CCT of 5462 K. For any constant ${\Delta \lambda }_{0}$, $\eta_{B}$ and $K_{B,v}$, depending on peak wavelength and CCT, can be described by sigmoidal functions.

Regarding pc-LEDs, a variation of both peak intensities, $S_{0}$ (blue LED) and $S_{\mathrm{ph}}$ (phosphor), resulted in a linear x-y-correlation, increasing bandwidths, ${\Delta \lambda }_{0}$ and ${\Delta \lambda }_{\mathrm{ph}}$, led to parabolic shaped x-y-curves accompanied by smaller x values, and changing $\lambda_{0}$ showed narrow parabola-like x-y-data, whereas $\lambda_{\mathrm{ph}}$ variations yielded broad x-y-patterns. $\eta_{B}$ and $K_{B,v}$ both increased and shifted to higher CCT with growing blue LED signal intensity, $S_{0}$, or decreasing phosphor emission, $S_{\mathrm{ph}}$, coinciding with most of the results for a ${\Delta \lambda }_{0}$ change. Exploiting the minima and avoiding the maxima of the curved CCT dependent $\eta_{B}$ and $K_{B,v}$, that appeared upon varying phosphor excitation bandwidth and both peak intensities, might be used to minimize pc-LEDs’ BLH risk concurrently preserving the desired CCT.

A comparison of the analytical $\eta_{B}$ and $K_{B,v}$ solutions with experimental values revealed that red, orange, and yellow LEDs are within 5% deviation. Presumably due to the spectroradiometer’s noise, $K_{B,v}$ for blue and green LEDs as well as $\eta_{B}$ for green LEDs are less accurate. For most of the pc-LEDs, the percentage deviations are within 0 to 7%.

Finally, the use of analytical BLH equations for color LEDs, the possibility to exploit the CCT dependence extrema of $\eta_{B}$ and $K_{B,v}$ for future pc-LEDs with less BLH, and the accuracy of the calculated data demonstrated the suitability of Gaussian functions to simulate LED emission spectra; thus, supporting risk assessment. However, this calculative approach has not been tested for tri- or tetra-chromatic LEDs, for arrays superimposing warm and cold pc-LEDs, or for organic LEDs. In addition to the most frequently used YAG phosphors, several others exist, and for many of them it is thought that their emitted light can be approximated by Gaussian functions, too, because of the fundamental physics of excitation and emission. However, no such phosphors have been examined for the present work. Furthermore, parameter variations influencing maximum permissible exposure durations have also not been addressed, yet, and the introduced Gaussian approach has not been compared to additional metrological and mere calculative BLH evaluation methods. Overall, this lack of knowledge provides work for future research.

## Figures and Tables

**Table 1:** Maximum relative spectral effectiveness, $A_{k}$, peak wavelength, $\lambda_{k}$, and bandwidth, $w_{k}$, according to the approximation of the blue-light hazard action spectrum in Equation (2) for the running index k= 1 to 5, reproduced from [24]. Least-squares fitting results for V(λ) by means of 4 Gaussian curves according to Equation (2) are given by k= 6 to 9. The parameter uncertainties are listed, too.

k	$A_{k}$	$\lambda_{k}\;(\mathrm{nm})$	$w_{k}\;(\mathrm{nm})$
1	0.2361	416.136	20.276
2	0.4443	423.378	215.925
3	0.8606	447.663	804.406
4	0.1505	480.662	118.811
5	0.0908	471.588	2697.525
6	0.895 ± 0.007	566 ± 1	3422 ± 22
7	0.23 ± 0.02	530.5 ± 0.2	1190 ± 30
8	0.023 ± 0.003	455 ± 1	576 ± 30
9	0.00014 ± 0.0011	712 ± 36	2000 ± 6000

**Table 2:** Experimental LED data used to test the accuracies of Equations (6) and (9). The running index j is assigned to each LED listed by its name, manufacturer, and label. The center wavelength of color LEDs, $\lambda_{c}\;$ according to Equation (11), and both experimental parameter triples are given in nm. $T_{\mathrm{cp}}$ (in K) is determined from the measured LED spectral irradiances.

j	Name (Manufacturer)	Label	$\lambda_{c}$	$\lambda_{0}\left|\Delta \lambda_{0}\right|S_{0}$	$\lambda_{\mathrm{ph}}\left|\Delta \lambda_{\mathrm{ph}}\right|S_{\mathrm{ph}}$	$T_{\mathrm{cp}}$
1	Diamond Dragon, W5AP (OSRAM)	LUW		451|25|1	551|131|0.41	9061
2		LCW		456|33|0.66	589|154|1	3403
3		LB	464	462|27|1		
4		LB #2	464	462|26|1		
5		LT #2	527	523|38|1		
6		LT	531	529|44|1		
7		LR	636	639|17|1		
8	ELS (Roithner)	blue	466	464|22|1		
9		green	519	515|33|1		
10		amber	595	596|14|1		
11		red	626	629|16|1		
12	Golden Dragon (OSRAM)	LUW W5KM		438|23|1	550|127|0.35	8487
13		LUW W5SM		442|27|1	551|128|0.40	9573
14		LUW W5SM #2		446|30|1	550|130|0.32	24,140
15		LW W51M		453|24|1	562|132|0.55	5770
16		ZW W5SG		454|25|1	563|130|0.48	6246
17		LCW W5SM #2		455|27|0.79	591|129|1	3168
18		LCW W5SM		456|28|0.78	590|129|1	3198
19		LW W55M		456|26|1	562|135|0.38	8458
20		LCW W55M		459|29|1	597|161|0.98	3514
21		LCW W51M		461|26|0.46	597|160|1	3097
22		LB W5SM	465	464|29|1		
23	Golden Dragon Plus, W5AM (OSRAM)	LUW		441|25|1	551|127|0.44	7854
24		LW		452|28|1	560|130|0.44	7953
25		LY	596	598|15|1		
26		LA	622	626|17|1		
27		LR	636	639|19|1		
28	HP 803 (Roithner)	NW		460|28|1	553|135|0.30	16,914
29		NB	469	469|34|1		
30		NR	630	636|15|1		
31	MR-16 (Omnilux)	cool white		450|27|1	556|127|0.46	7698
32		warm white		460|28|0.55	582|105|1	3460
33		blue	461	461|29|1		
34		green	523	520|36|1		
35		yellow	597	600|16|1		
36		red	636	641|18|1		
37	Paulmann	LED-Reflector		450|24|1	557|124|0.55	6051
38	Platinum Dragon, W5SN (OSRAM)	LW		452|31|1	564|133|0.62	5943
39		LCW		458|26|0.93	589|129|1	3314
40		LA	625	628|17|1		
41		LR #2	631	635|17|1		
42		LR	633	637|18|1		

## Data Availability

The data presented in this study are available on request from the corresponding author.

## Appendix A

### Appendix A.1. Chromaticity Coordinates

Chromaticity coordinates, (x, y, z), are very useful regarding the color definition of light sources or reflecting surfaces [38]. Calculating (x, y, z) is a well-known procedure and is standardized, for example, by ISO/CIE [39]. Briefly summarizing the basic concept, the signal S(λ) is convoluted with each of the three color-matching functions of the CIE 1931 2° standard colorimetric observer, $\left(\bar{x,}\bar{y,}\bar{z}\right)$. These functions were derived by a linear transformation of the experimentally determined color-matching stimuli for green, blue, and red, because the latter contains undesired negative values. Due to the dependence on the observer’s view, there also exist $\left(\bar{x},\;\bar{y},\;\bar{z}\right)$ for a CIE 1964 10° standard observer (not used in this work). Multiplying the convolution by $K_{m}$ yields the tristimulus values.

(A1) $$\left(\begin{array}{c} X\\ Y\\ Z \end{array}\right)=K_{m}\overset{830\;\mathrm{nm}}{\underset{360\;\mathrm{nm}}{\int }}S\left(\lambda \right)\;\left(\begin{array}{c} \bar{x}\\ \bar{y}\\ \bar{z} \end{array}\right)\left(\lambda \right)\;d\lambda$$

Note the integration limits running from 360 to 830 nm in contrast to the BLH spectral region, 300 to 700 nm, or the visible wavelength range, 380 to 780 nm. Normalizing (X, Y, Z) results in relative magnitudes called the chromaticity (or color) coordinates.

(A2) $$\left(\begin{array}{c} x\\ y\\ z \end{array}\right)=\frac{1}{X+Y+Z}\left(\begin{array}{c} X\\ Y\\ Z \end{array}\right)$$

With x+y+z= 1, it is sufficient to specify color by only two variables, typically (x, y), providing the basis for two-dimensional chromaticity diagrams.

The “color” of white-light sources is also expressed by chromaticity coordinates, usually referred to as white points. For example, the theoretical equal energy illuminant E with its constant emission signal has the white point x=y= ⅓, and the chromaticity coordinates for the daylight illuminant D65, part of CIE’s standard illuminant series D, are given by x= 0.31273 and y= 0.32902. In addition to the color definition, chromaticity coordinates can also be applied to calculate the correlated color temperature (CCT) of light sources.

### Appendix A.2. Correlated Color Temperature

The spectral radiation emission of a blackbody radiator is determined by its absolute temperature, T, via

(A3) $$S\left(\lambda ,T\right)=2\pi hc^{2}\lambda^{-5}{\left[\exp\left(\frac{hc}{\lambda \;k_{B}\;T}\right)-1\right]}^{-1}$$

with h= 6.626 × 10^−34^ Js (Planck’s constant), c= 3 × 10^9^ ms^−1^ (speed of light), and $k_{B}=$ 1.38 × 10^−23^ JK^−1^ (Boltzmann’s constant). For any light source with its chromaticity coordinates being close to the Planckian (blackbody) locus, absolute and color temperature match each other. If (x, y, z) are not equal, the CCT, $T_{\mathrm{cp}}$, refers to the absolute temperature of the blackbody radiator whose chromaticity coordinates are nearest to that of the light source in a uniform chromaticity scale diagram. Apart from several algorithms to find this smallest off locus colorimetric distance, McCamy [40] derived a $T_{\mathrm{cp}}$ formula using a cubic polynomial,

(A4) $$T_{\mathrm{cp}}=an^{3}+bn^{2}+cn+d$$

with parameters a= −437 K, b= 3601 K, c= −6861 K, and d= 5514.31 K. The inverse isotemperature line slope n is given by

(A5) $$n=\frac{x-x_{e}}{y-y_{e}}$$

and the $T_{\mathrm{cp}}$ epicenter is located at $x_{e}=$ 0.3320 and $y_{e}=$ 0.1858. While McCamy’s equation is restricted to CCT values 2000 K $\leq T_{\mathrm{cp}}\leq$ 12,500 K, Hernández-Andrés et al. [41] could increase the $T_{\mathrm{cp}}$ range up to $8\times 10^{5}$ K by using a sum of exponential functions.

(A6) $$T_{\mathrm{cp}}=A_{0}+A_{1}\exp\left(-\frac{n}{t_{1}}\right)+A_{2}\exp\left(-\frac{n}{t_{2}}\right)+A_{3}\exp\left(-\frac{n}{t_{3}}\right)$$

There are two sets of parameters $A_{i}$ and $t_{i}$, one for 3000 K $\leq T_{\mathrm{cp}}\leq$ 50,000 K and another one for 50,000 K $\leq T_{\mathrm{cp}}\leq$ 8 $\times 10^{5}$ K. The inverse slope n is defined in accordance to Equation (A5) but with different CCT epicenters, $x_{e}$ and $y_{e}$. It is worth mentioning that “there have been reports that CCT is not appropriate for a source like LEDs due to their spectral characteristics” [15]. A comparison not only of Equations (A4) with (A6) but also with additional CCT computation methods can be found in a publication by Li et al. [42].
